# CRISPR-Mediated Strand Displacement Logic Circuits with Toehold-Free DNA

**DOI:** 10.1021/acssynbio.0c00649

**Published:** 2021-04-26

**Authors:** Roser Montagud-Martínez, María Heras-Hernández, Lucas Goiriz, José-Antonio Daròs, Guillermo Rodrigo

**Affiliations:** † I2SysBio, CSIC − Universitat València, Cat. Agustín Escardino 9, 46980 Paterna, Spain; ‡ IBMCP, CSIC − Universitat Politècnica València, Av. Naranjos s/n, 46022 Valencia, Spain

**Keywords:** biological computing, DNA nanotechnology, synthetic biology

## Abstract

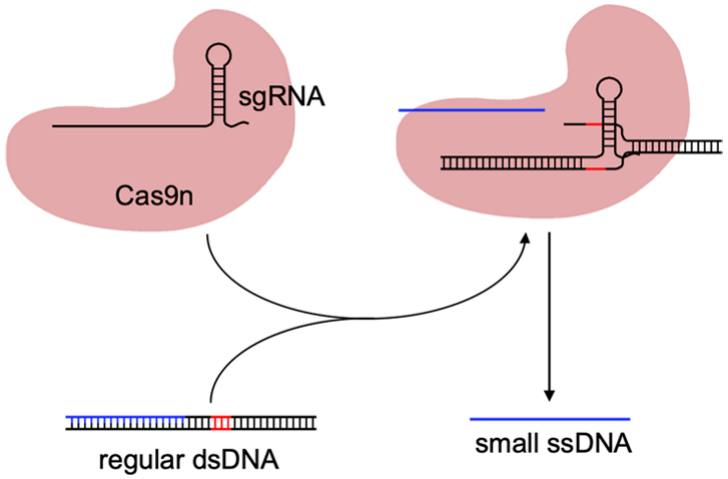

DNA nanotechnology, and DNA computing in particular, has grown extensively over the past decade to end with a variety of functional stable structures and dynamic circuits. However, the use as designer elements of regular DNA pieces, perfectly complementary double strands, has remained elusive. Here, we report the exploitation of CRISPR-Cas systems to engineer logic circuits based on isothermal strand displacement that perform with toehold-free double-stranded DNA. We designed and implemented molecular converters for signal detection and amplification, showing good interoperability between enzymatic and nonenzymatic processes. Overall, these results contribute to enlarge the repertoire of substrates and reactions (hardware) for DNA computing.

Apart from being at the ground of all known autonomous forms of life,^1^ deoxyribonucleic acid (DNA) is a unique substrate from which to build sophisticated molecular programs that can run *in vitro*.^2−5^ Such programs are typically implemented through the conditional assembly of single-stranded DNA (ssDNA) species *via* Watson–Crick base pairing, which allows sensing and releasing different strands. Yet, ribonucleic acid (RNA) can also be at play due to its ability to interact with DNA to form hybrid species.^6^ Briefly, DNA strand displacement works thanks to a toehold^7^ (an overhanging region), which triggers the branch migration process of an invading ssDNA species over a double-stranded DNA (dsDNA) molecule to end in a more stable conformation. According to this mechanism, however, the use of regular dsDNA (*i.e*., dsDNA with blunt ends) has been excluded from this framework because of the intrinsic absence of toeholds in these molecules. Hence, regular dsDNA species have constrained activity in current DNA circuits.

Beyond the initial development of toehold-mediated strand displacement,^7^ different variants have been proposed in order to increase functionality. For example, the insertion of a variable spacer between the toehold and displacement domains allows tuning the reaction rate,^8^ and toehold switching is possible if these domains belong to different strands that are hybridized through a third region.^9^ Furthermore, to avoid the output of regular dsDNA species (and then waste material), systems with toehold exchange were developed.^10,11^ That is, systems in which the invading strand is not fully complementary to its target and the resulting dsDNA molecule has a novel toehold in the opposite end. Intriguingly, entropy drives these reactions, which allows decoupling thermodynamics and kinetics.^11^

In recent work, DNA circuits have been expanded thanks to the action of particular enzymes, such as nicking endonucleases^12,13^ and DNA polymerases.^13,14^ Certainly, the introduction of enzymes can increase the performance of the intended behavior, such as to recycle output products^12^ or to enhance the detection limit of the input molecule much below the nanomolar scale.^13^

In this communication, we introduce the concept of CRISPR-mediated strand displacement in order to work with regular dsDNA in logic circuits (CRISPR stands for clustered regularly interspaced short palindromic repeats);^14^ that is, to exploit as functional elements, rather than being mere waste products, dsDNA molecules that lack toeholds. For that, we used a CRISPR-associated 9 (Cas9) protein to, in combination with appropriately designed small guide RNAs (sgRNAs), target specific DNA sequences.^15^ In particular, we based our circuits on the *Streptococcus pyogenes* Cas9, but nothing prevents the use of other CRISPR proteins able to target DNA, such as the *Acidaminococcus sp.* Cas12a.^16^ In particular, we harnessed a partially catalytically inactive form working like a nickase (written as Cas9n).^15^ This variant has the H840A mutation (in the HNH domain), which disables the cleavage on the target strand (where the sgRNA binds). The nontarget strand is cleaved 3 nt upstream the protospacer adjacent motif (PAM) sequence. Interestingly, when a PAM sequence is close to an end of the dsDNA fragment (let us say between 17 and 40 bp), the CRISPR-Cas9n system can be programmed to produce an ssDNA molecule directly from the nontarget strand.

We proved the suitability of this approach by engineering different logic circuits responsive to toehold-free dsDNA molecules, producing as outputs individual oligonucleotides. These circuits worked isothermally. Fluorescence and gel electrophoresis assays were instrumental to get mechanistic insight about the functioning.

We started with the engineering of a molecular converter from dsDNA to ssDNA species (Figure 1a). Here, we designed the sgRNA *GUI1* (with a protospacer of 20 nt) to produce the ssDNA *OUT1* (of 17 nt) from a regular dsDNA piece of 36 bp (*IN1*; sequences shown in Table S1). Because the nontarget strand has 3D contacts with the Cas9 protein,^17,18^ the excised fragment remains bound to the complex and cannot be released to the medium in a spontaneous manner.^19^ Thus, we added proteinase K after completing the CRISPR reaction to rescue *OUT1*, in order to be the input in subsequent downstream reactions (Figure 1b). By placing the 6-carboxyfluorescein fluorescent dye in the 5′ end of *IN1* and the Iowa Black FQ dark quencher in the cognate 3′ end,^20^ we were able to measure the displacement of *OUT1*. The fluorescence results revealed a significant performance, with an efficiency of 71.2% (using as a reference the maximal dynamic range related to the free and quenched dye) and no apparent basal release in absence of sgRNA or Cas9n (Figure 1c). These reactions occurred isothermally at 37 °C (Cas9n:sgRNA:DNA ratio of about 5:15:1, noting that the sgRNA by itself cannot produce the displacement of *OUT1*, even at a high concentration). Next, we assayed the system by nondenaturing polyacrylamide gel electrophoresis (PAGE), staining with silver, to confirm the release of *OUT1* from *IN1* (Figure 1d).

**Figure 1:**
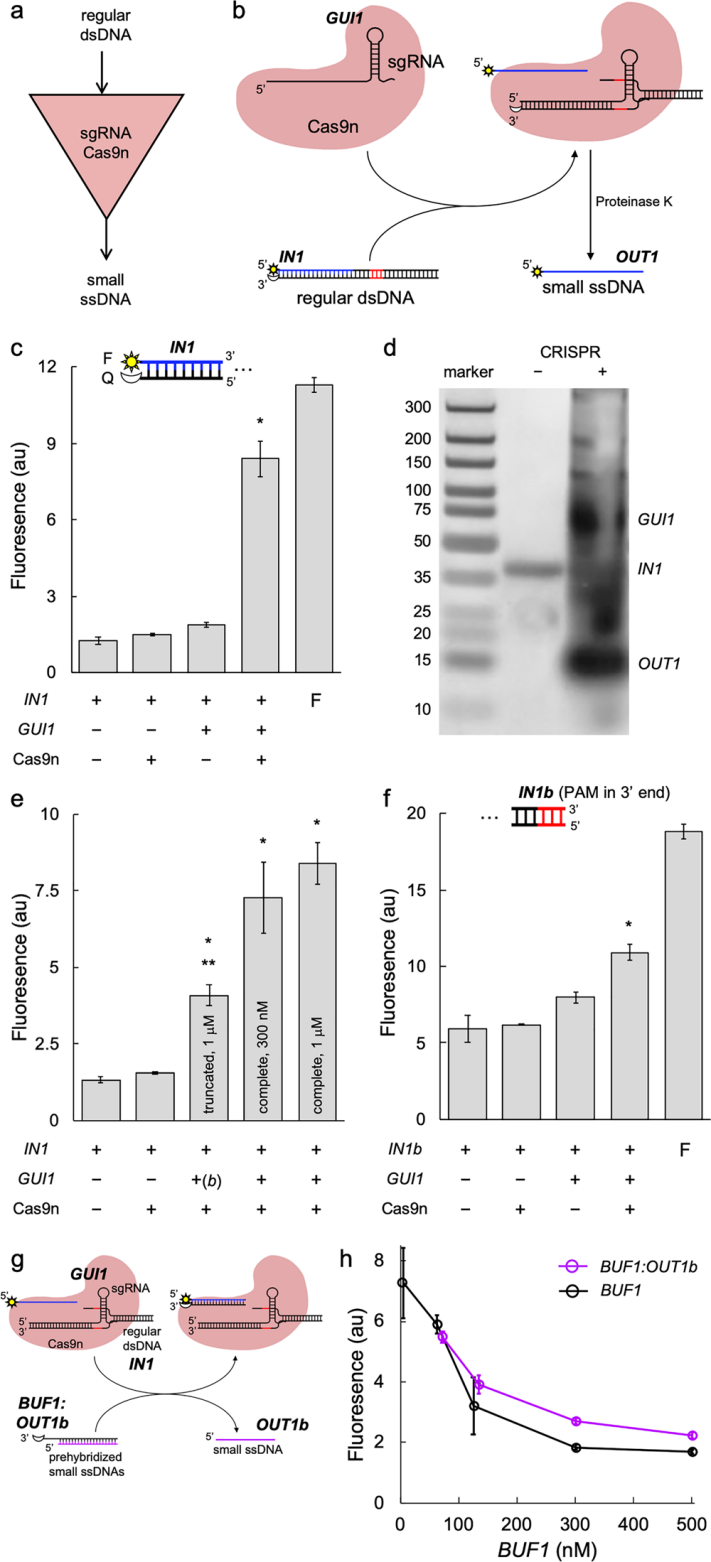
Engineering a molecular converter based on CRISPR-mediated DNA strand displacement. (a) Logic scheme of the biochemical reaction. (b) Implementation of the reaction by exploiting a CRISPR-Cas system *in vitro*. The excised strand is marked in blue and the PAM sequence in red. (c) Characterization of the intended strand displacement (in panel b) by using a fluorophore (F, sun icon) and a quencher (Q, moon icon). F bar corresponds to a single oligo labeled with the fluorophore. *IN1* at 62.5 nM, *GUI1* at 1 μM, and Cas9n at 300 nM. (d) Electrophoretic assay to confirm the release of the ssDNA after a treatment with proteinase K. The different species of the system are indicated. (e) Assessment of the sgRNA effect in terms of sequence and concentration (sgRNA from 1 μM to 300 nM). (f) Assessment of the PAM position effect. F bar corresponds to a single oligo labeled with the fluorophore. (g) Implementation of an alternative CRISPR reaction to produce strand displacement from regular dsDNA. (h) Characterization of the intended strand displacement (in panel g) for different concentrations of *BUF1* (either prehybridized with another oligo or alone). *IN1* at 62.5 nM, *GUI1* at 300 nM, and Cas9n at 300 nM. Error bars correspond to standard deviations over replicates (*n* = 3). Statistical significance (Welch’s *t*-test, two-tailed *P* < 0.05) of higher fluorescence with respect to any of the negative controls (*) and lower fluorescence with respect to the system with complete sgRNA (**).

To inspect this process in more detail, we performed nested enzymatic reactions with proteinase K, ribonuclease (RNase) A, and RNase H (Figure S1a). Our results showed that the sgRNA remains bound to the nicked dsDNA molecule after removal of Cas9n, and that this resulting hybrid species (RNA-DNA) is instrumental to prevent the return of the output ssDNA molecule to reconstitute the input element (Figure S1b).

We also found that if the sgRNA is truncated by removing the transcriptional terminator (from *S. pyogenes*; resulting in *GUI1b*), the system significantly loses efficiency (Figure 1e). In particular, it decreases from 71.2% to 28.2%. This agrees with the fact that there are 3D contacts between the terminator and Cas9,^17,18^ pointing out that the formation of the ribonucleoprotein is compromised in this case. However, when the concentration of the sgRNA is reduced to the same level of Cas9n (leading to a Cas9n:sgRNA:DNA ratio of about 5:5:1), the system still works with substantial efficiency, as expected from the fact that the ribonucleoprotein is formed efficiently. We further found that if the PAM sequence is located in the very 3′ end of the input dsDNA molecule (*IN1b*), the ribonucleoprotein only performs with an efficiency of 38.9% (Figure 1f).

In addition, we investigated the possibility to produce strand displacement from regular dsDNA molecules by avoiding the degradation of Cas9n by proteinase K. For that, we hypothesized that the resulting CRISPR complex after targeting might displace a prehybridized strand in a toehold-mediated manner (Figure 1g), as previous work has pointed out that ssDNA species can interact with the nontarget strand.^19,21^ Using *IN1* as trigger dsDNA molecule, our results revealed that an ssDNA species in a complex (*OUT1b*) can be released to the medium through a CRISPR reaction (Figure 1h). However, the relative amount of *BUF1*:*OUT1b* (with respect to *IN1*) needs to be high for an efficient displacement.

Subsequently, we engineered a molecular amplifier based on reactions of DNA strand displacement, combining CRISPR-mediated with toehold-mediated reactions (Figure 2a). In particular, we implemented a 2-fold signal amplification (*i.e.*, one input ssDNA molecule leads to two output ssDNA molecules). For that, we thought to exploit the regular dsDNA molecule that is produced in a conventional toehold-mediated strand displacement reaction as an intermediate species thanks to a given sgRNA and Cas9n (Figure 2b). In electronic terms, this would result in a close-loop amplification scheme, as the first-instance output is recycled in the system.

**Figure 2:**
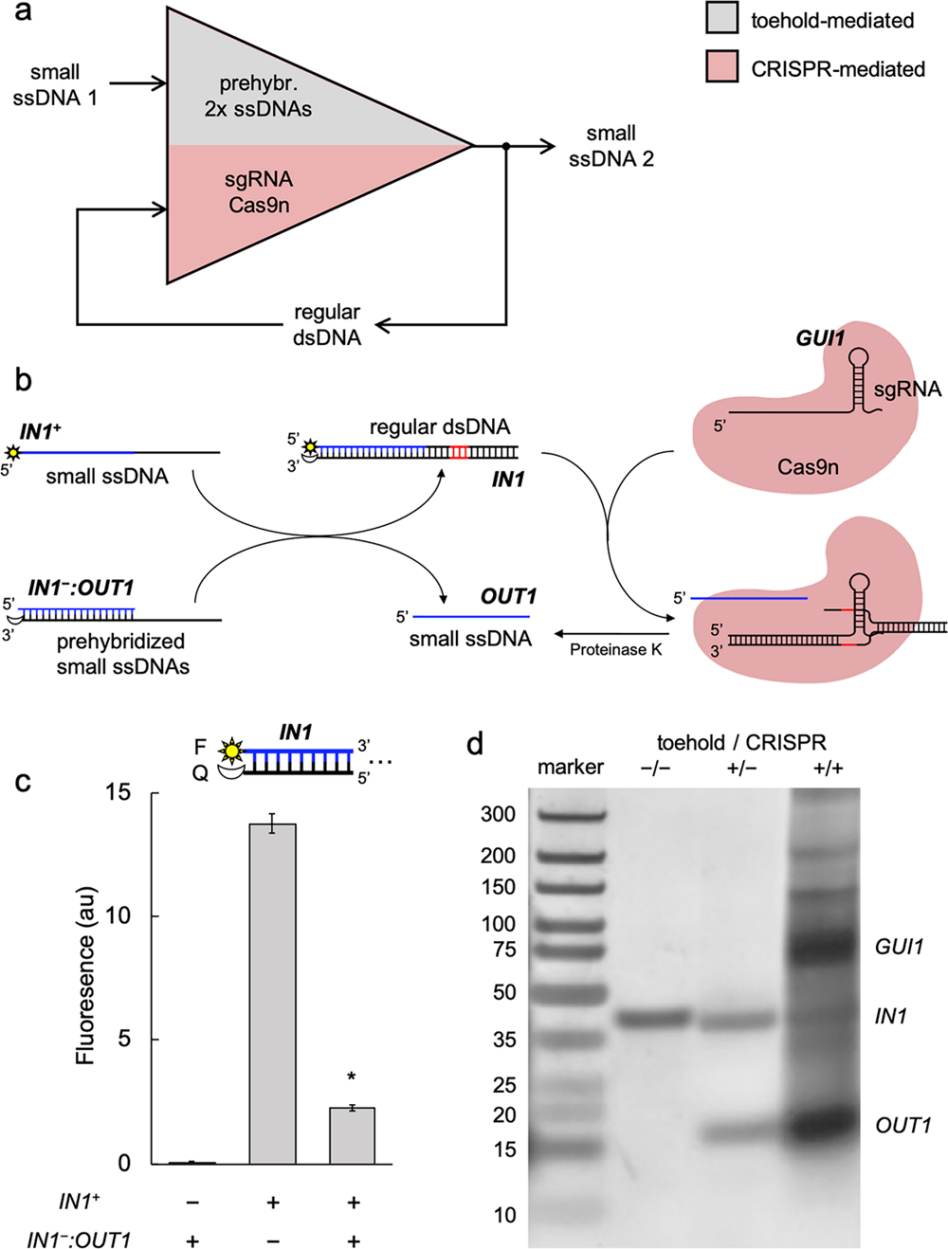
Engineering a close-loop molecular amplifier based on CRISPR- and toehold-mediated DNA strand displacement. (a) Logic scheme of the biochemical reactions. (b) Implementation of the reactions by exploiting a CRISPR-Cas system *in vitro*. The displaced/excised strand is marked in blue and the PAM sequence in red. (c) Characterization of the toehold-mediated strand displacement by using a fluorophore (F, sun icon) and a quencher (Q, moon icon). *IN1*^*+*^ at 62.5 nM, *IN1*^–^:*OUT1* at 62.5 nM, *GUI1* at 300 nM, and Cas9n at 300 nM. Error bars correspond to standard deviations over replicates (*n* = 3). Statistical significance (Welch’s *t*-test, two-tailed *P* < 0.05) of lower fluorescence with respect to the positive control (*). (d) Electrophoretic assay to confirm the signal amplification after a treatment with proteinase K. The different species of the system are indicated. In lane −/–, the band corresponds to *IN1*^–^:*OUT1*.

Specifically, we took advantage of the previous CRISPR-based system (production of *OUT1* from *IN1*) to engineer our amplifier. By writing *IN1* as *IN1*^*+*^:*IN1*^*–*^ (*i.e*., considering each strand as an individual ssDNA species), the reaction *IN1*^*+*^ (input) plus *IN1*^*–*^:*OUT1* (gate) is mediated by a toehold of 19 nt and leads to *OUT1* (output) plus *IN1* (waste). Thus, by placing the fluorescent dye in the 5′ end of *IN1*^*+*^ and the dark quencher in the 3′ end of *IN1*^*–*^, we were able to measure the release of *OUT1* by fluorescence suppression (Figure 2c), showing an efficiency of 92.7%. Next, we introduced into the system the sgRNA *GUI1* and Cas9n, expecting the subsequent processing of *IN1* to generate an additional molecule of *OUT1*. Potential interferences between the two reactions are limited because no PAM sequence exists in *IN1*^*–*^:*OUT1.* As before, these reactions occurred isothermally at 37 °C. To confirm the intended amplification, we assayed the system by nondenaturing PAGE, staining with silver (Figure 2d). Band quantification with Fiji (a distribution of ImageJ)^22^ gave an amplifier gain of 2.91 (we attributed this value >2, at least in part, to working in a concentration regime close to the detection limit in silver-stained PAGE). Figure S2 shows a different gel in which RNase A was also added. We then concluded that CRISPR systems are able to recycle regular dsDNA products from toehold-mediated strand displacement reactions.

Motivated by these results, we decided to implement a cascade of strand displacement events in which the first event corresponds to a CRISPR reaction (Figure 3a). First, from a new toehold-free dsDNA piece of 46 bp (*IN2*), we designed an appropriate sgRNA (*GUI2*), with a protospacer of 25 nt, to produce the ssDNA *OUT2* (of 22 nt; sequences shown in Table S1). Second, we designed an interface based on toehold-mediated strand displacement to interconvert two arbitrary ssDNA species. Taking *OUT2* as the incoming element, the interface is implemented through a sensor molecule (*BUF2*) and a clamp molecule (*BUF3*) that are initially prehybridized with a transducer molecule (*BUF2b*) and the outcoming element (*OUT3*), respectively. This way, *OUT2* can interact with *BUF2* through a toehold of 6 nt to release *BUF2b*, which in turn can interact with *BUF3* through a now exposed toehold of also 6 nt to release *OUT3* (Figure 3b; see also Figure S4a). We implemented a small algorithm (in Python) to perform the automated sequence design of the species *BUF2*, *BUF2b*, and *BUF3*, provided the sequences of *OUT2* and *OUT3* (Figure S3).

**Figure 3:**
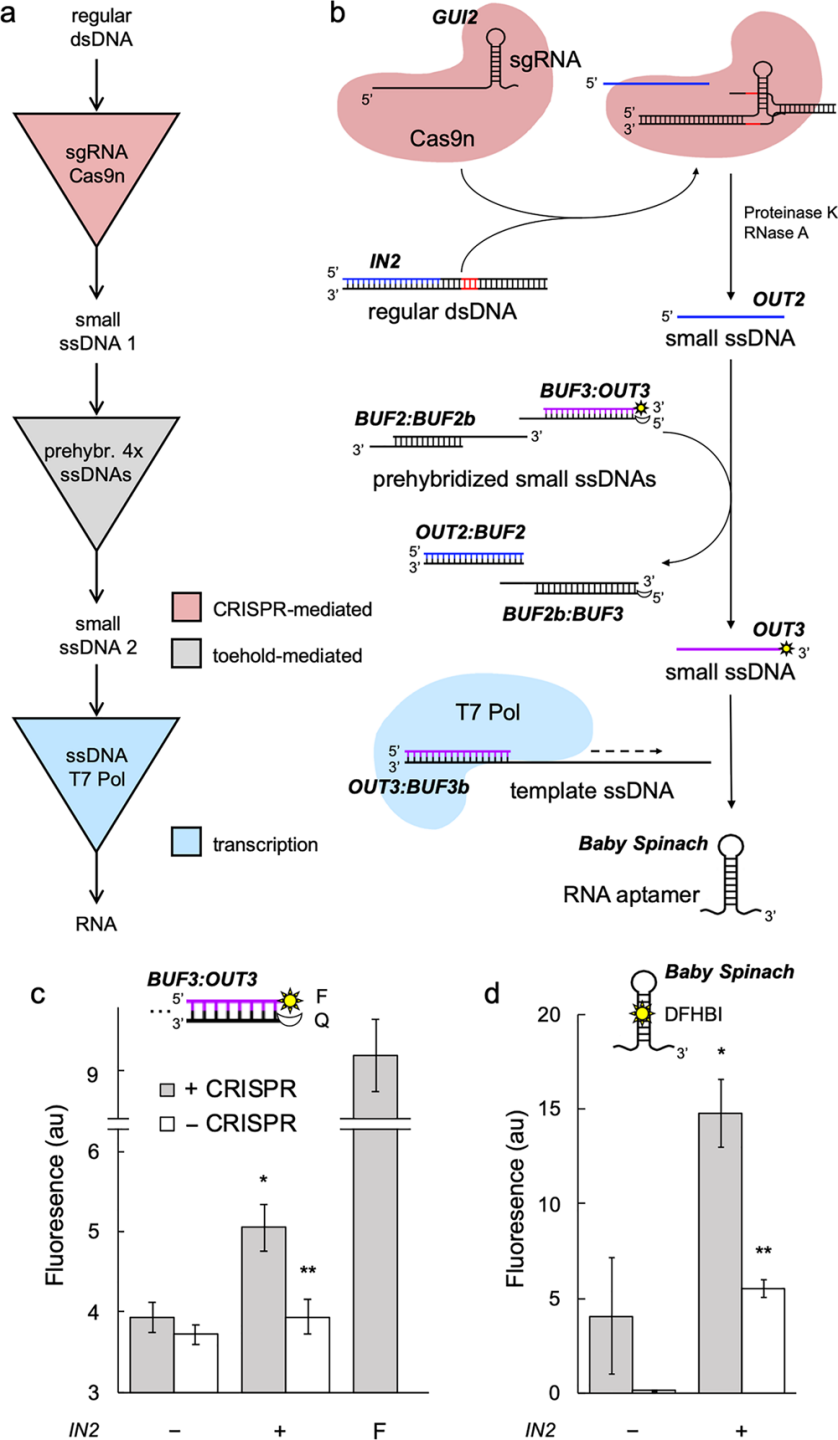
Engineering a serial cascade based on CRISPR- and toehold-mediated DNA strand displacement and *in vitro* transcription. (a) Logic scheme of the biochemical reaction. (b) Implementation of the reaction by exploiting a CRISPR-Cas system *in vitro*. The excised strand is marked in blue, the PAM sequence in red, and the output displaced strand of the second step in purple. (c) Characterization of the intended strand displacement by using a fluorophore (F, sun icon) and a quencher (Q, moon icon). F bar corresponds to a single oligo labeled with the fluorophore. *IN2* at 125 nM, *GUI2* at 600 nM, Cas9n at 600 nM, *BUF2:BUF2b* at 62.5 nM, and *BUF3:OUT3* at 15.6 nM. (d) Characterization of the whole cascade, including a step of *in vitro* transcription, with the fluorescent aptamer upon addition of DFHBI. *BUF3b* at 7 nM. Error bars correspond to standard deviations over replicates (*n* = 3). Statistical significance (Welch’s *t*-test, two-tailed *P* < 0.05) of higher fluorescence with input (*) and lower fluorescent with respect to the +CRISPR condition (**).

Experimentally, we first incubated the CRISPR step with the input dsDNA molecule. Then, we added one at a time proteinase K (to digest Cas9n), phenylmethylsulfonyl fluoride (PMSF, to inactivate proteinase K), and RNase A (to digest the sgRNA). Next, the prehybridized complexes *BUF2*:*BUF2b* and *BUF3*:*OUT3* were introduced. Here, the fluorescent dye was placed in the 3′ end of *OUT3* and the dark quencher in the 5′ end of *BUF3*. The whole reaction run isothermally at 37 °C. Our results showed the release of *OUT3* in response to *IN2*, with an efficiency of 21.3% (Figure 3c). They also confirmed that in absence of CRISPR species the reaction does not progress. We further assessed such a release in response to *OUT2* (Figure S4b), with an efficiency of 34.7%, and *BUF2b* (Figure S4c), although with other concentrations of the species. We hence concluded that the output of a CRISPR-mediated strand displacement reaction can act as the input of a downstream toehold-mediated reaction.

Because there is freedom to choose the element *OUT3*, we designed it to be the forward sequence of a T7 promoter. This way, *OUT3* can be exploited to produce functional RNAs through a subsequent step of *in vitro* transcription with the T7 RNA polymerase, provided a template strand is added to the medium (*BUF3b*). The use of ssDNA species to reconstitute T7 promoters has been already employed to engineer dynamic circuits *in vitro*.^23^ Here, we decided to express the RNA aptamer Baby Spinach.^24^ We chose this aptamer because it is a miniaturized aptamer with good fluorescent properties, but nothing prevents the use of other aptamers, such as Broccoli^25^ or Mango.^26^ In addition, we anticipate that the resulting transcript might also act in future developments as a new RNA species to trigger further strand displacement reactions, or even be a new sgRNA. Notably, we found that our cascade formed by an initial step of CRISPR-mediated strand displacement, an intermediate step of toehold-mediated strand displacement, and a final step of *in vitro* transcription, monitored through the addition of 3′5′-difluoro-4-hydroxybenzylidene imidazolinone (DFHBI), is fully functional (Figure 3d).

It is worth to note at this point that the transducer element (*BUF2b*), as it shares sequence with *OUT3*, might reconstitute a functional T7 promoter upon interaction with *BUF3b* in absence of input (note that both *OUT3* and *BUF2b* end in TATAGG). Consequently, we designed *BUF2b* to accommodate a mutation (C to T in the −7 position of the promoter)^27^ that weakens the transcriptional activity, which allowed us to obtain reasonable results (Figure S4d). We tried to further reduce the leakage by introducing other mutations in *BUF2b* according to previous work (*e.g.*, C to A in that −7 position),^27^ but we did not succeed.

Finally, we engineered a combinatorial device by combining CRISPR-mediated with toehold-mediated reactions (Figure 4a). In this case, two different molecules (one ssDNA, *IN4*, and one dsDNA, *IN5*) work together to release the output element (*OUT6*; sequences shown in Table S1). First, we designed an appropriate sgRNA (*GUI5*), with a protospacer of 41 nt, to produce the ssDNA *OUT5* (of 38 nt) from *IN5*. Second, we designed a complex of three prehybridized ssDNAs (*BUF4*:*BUF5*:*OUT6*, AND gate element) to trap the output molecule in a conditional way. For that, we took advantage of previous work on enzyme-free DNA logic circuits.^4^ Initially, the gate is only sensitive to *IN4*, which invades it through a toehold of 6 nt to remove *BUF4*. As a result, *BUF5*:*OUT6* is sensitive to *OUT5*, which with a toehold of also 6 nt located in its 3′ end to interact with *BUF5* allows the release of *OUT6* (Figure 4b).

**Figure 4:**
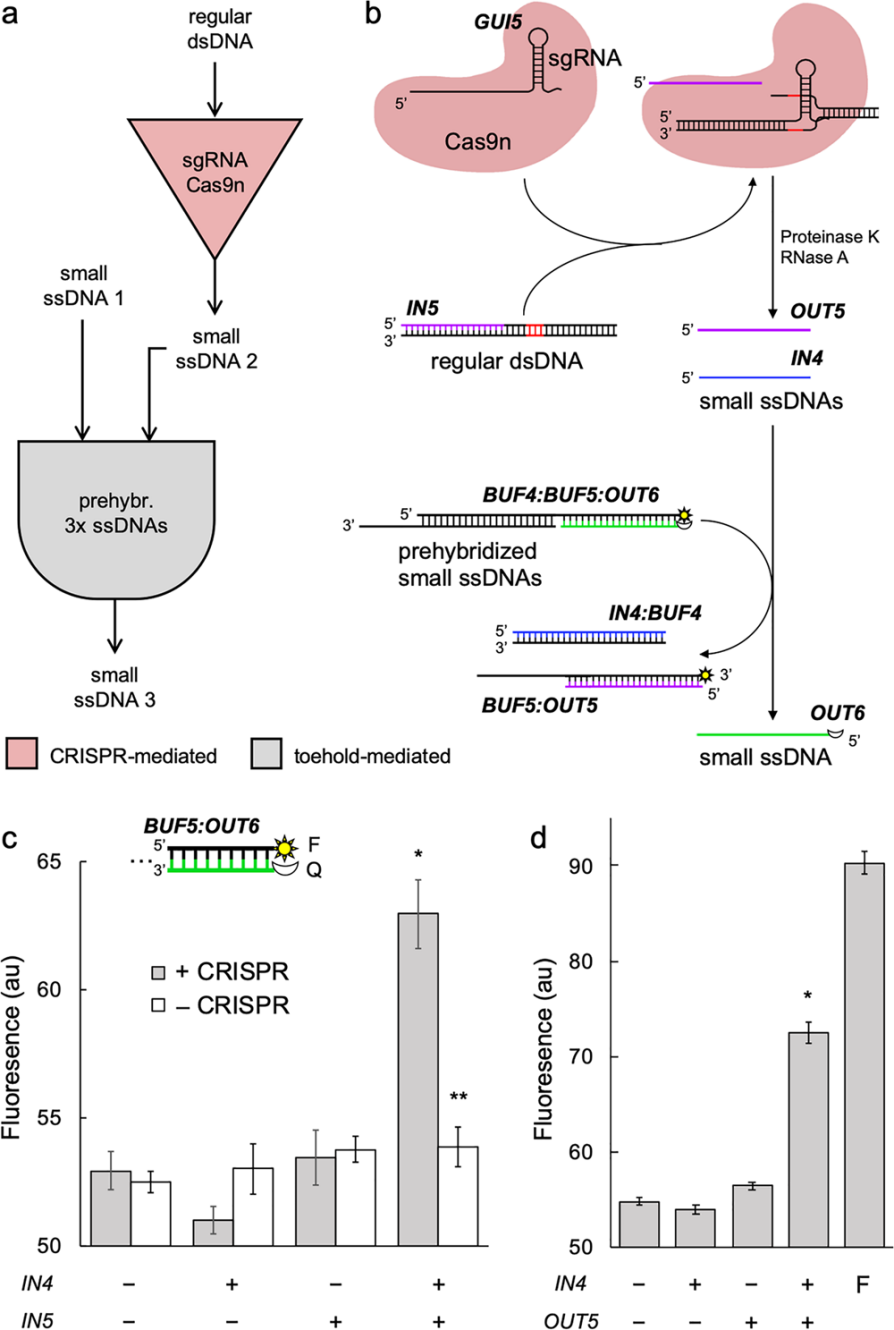
Engineering a combinatorial device working as an AND gate based on CRISPR- and toehold-mediated DNA strand displacement. (a) Logic scheme of the biochemical reactions. (b) Implementation of the reactions by exploiting a CRISPR-Cas system *in vitro*. The excised strand is marked in purple (the other input strand in blue), the PAM sequence in red, and the output displaced strand of the second step in green. (c) Characterization of the intended strand displacement by using a fluorophore (F, sun icon) and a quencher (Q, moon icon). *IN4* at 62.5 nM, *IN5* at 125 nM, *GUI5* at 600 nM, Cas9n at 600 nM, gate at 62.5 nM. (d) Characterization of the toehold-mediated strand displacement (gate) with the ssDNA species. F bar corresponds to a single oligo labeled with the fluorophore. Error bars correspond to standard deviations over replicates (*n* = 3). Statistical significance (Welch’s *t*-test, two-tailed *P* < 0.05) of higher fluorescence with two inputs (*) and lower fluorescence with respect to the +CRISPR condition (**).

To implement this reaction, we first incubated the CRISPR step, with the ssDNA and dsDNA inputs and the CRISPR species. Then, we added one at a time proteinase K, PMSF, and RNase A. Subsequently, we added the gate. To assess the performance of the system, the fluorescent dye was placed in the 3′ end of *BUF5* and the dark quencher in the 5′ end of *OUT6*. The whole reaction was isothermal at 37 °C. Our results showed the synergistic release of *OUT6* by the action of *IN4* and *IN5*, with an efficiency of 28.3% (with respect to the maximal dynamic range between the free and quenched dye) and no apparent basal release in absence of the CRISPR ribonucleoprotein (Figure 4c; we attributed the high background fluorescence to a modest quenching and a partial dehybridization of the AND gate). This encourages the future development of more complex programs^4,28^ with regular dsDNA molecules.

To independently verify the functioning of the toehold-mediated AND gate, we synthesized the oligonucleotide *OUT5* to serve as a direct input in the reaction. This way, we assayed the response of the gate *BUF4*:*BUF5*:*OUT6* to the species *IN4* and *OUT5*, finding similar results as in the case of the system including the CRISPR step, now with an efficiency of 47.1% (Figure 4d). This indicated that the enzymatic and nonenzymatic reactions performed in a similar way.

We also observed that the sgRNA *GUI5* alone, without Cas9n, is able to interact with *BUF5* (as the protospacer of *GUI5* contains the RNA form of *OUT5*; Figure S5a) and then, in conjunction with *IN4*, activate the release of *OUT6* (Figure S5b). The presence of Cas9n, however, cuts off the activation, presumably due to the lack of a PAM sequence in the gate. Consequently, the use of RNase A to remove the different RNA species of the system seems instrumental to avoid false positives when combining both types of strand displacement reactions. Moreover, we tested if the species *BUF5*:*OUT6* is able to interact with *OUT5* (the nontarget strand) in the CRISPR complex, finding that, in the concentration regime employed, such an interaction is not produced (Figure S6).

In conclusion, this work originally shows that a given regular dsDNA fragment, without toehold, can be used as a substrate in strand displacement reactions to engineer logic circuits, provided elements of the CRISPR-Cas technology^14^ are added, thereby circumventing the fundamental design principle of this type of biocomputation.^7^ Yet, this development is straightly compatible with conventional systems based on strand displacement.^2−5^ In light of our results, CRISPR-mediated strand displacement leads to the generation of defined, individual ssDNA molecules, which can then trigger downstream nonenzymatic DNA reactions. In turn, dsDNA products from toehold-mediated strand displacement reactions might be recycled to the system through the use of CRISPR ribonucleoproteins, although this would require various steps with our current implementation. Excised ssDNA strands might contribute to amplify the output signal or to extend the cascade by interacting with further species.

We expect a wider catalogue (and a significant reduction in the price) of commercial CRISPR proteins in the coming years, which will allow a widespread use of these systems. The rational engineering of these proteins might lead to novel features, such as the ability to release the nontarget strand from a small dsDNA molecule in the case of Cas9. This would simplify the implementation of our circuits. Alternatively, Cas12a, which does release DNA after cleavage, might be exploited as a producer of dsDNA species with a toehold of 5 nt^16^ to be interfaced with downstream reactions. Certainly, strand displacement principles can be enlarged with the use of RNA-guided nucleases to lead to a new generation of engineered biodevices.

Importantly, the repurposing of CRISPR-based systems is already allowing the development of novel strategies for (pre)clinical diagnostics, such as to detect viral infections^29,30^ and to isothermally amplify DNA molecules.^21,31^ Of note, these systems have even been applied to detect SARS-CoV-2 in clinical samples in the current pandemic scenario.^32,33^ Our logic circuits might be of utility in this area as well, provided a preamplification process is applied. Certainly, conventional DNA circuits have been applied to sense microRNAs^4^ (potential markers of diseases in biological samples).^34^ Regular dsDNA fragments might also be exploited as biomarkers of certain diseases, such as some types of cancer, as they can freely circulate throughout the human body in the blood (with a size between 100 and 200 bp).^35^ We also anticipate that it might also be possible to generate a given ssDNA species from a long regular dsDNA molecule with the use of two different sgRNAs, ensuring that both nickases cleaved the same strand (and provided there were two PAM sequences flanking the intended region).^36^ If so, plasmids might also be directly used as inputs.

All in all, since a controlled strand displacement is the basis of promising molecular machines,^28^ an extension of the hardware (*i.e.*, the use of toehold-free dsDNA) is expected to significantly boost their programmability and functional sophistication in order to reach a variety of applications.

## Methods

### Reagents

The strand displacement reactions were carried out in 1× TAE buffer pH 8.5 (Invitrogen) supplemented with 12.5 mM MgCl_2_ (Merck) and 0.05% Tween 20 (Merck). The different oligos were chemically synthesized by Sigma (now Merck) or IDT. For CRISPR-mediated strand displacement, the *S.p*. Cas9 H840A Nickase V3 (IDT) was used. Additional enzymes and chemicals were used: proteinase K (Invitrogen), RNase A (Invitrogen), RNase H (Ambion), RNase inhibitor (Applied), PMSF (Thermo), and DFHBI (Merck).

### Reactions

All sgRNAs were produced by *in vitro* transcription (TranscriptAid T7 High Yield Transcription kit, Thermo) and then purified in a column (RNA clean and concentrator kit, Zymo). The CRISPR reactions were performed during 1 h, with the input species (dsDNA or ssDNA) at 62.5–125 nM, sgRNA at 300–1000 nM, and Cas9n at 300–600 nM (precise concentrations specified in any case). To release the nicked ssDNA, the resulting products were treated in the same tube with proteinase K (200 μg/mL) for 30 min, then with PMSF (1 mM) for 30 min, and then with RNase A (20 μg/mL) for 30 min. All steps were carried out isothermally at 37 °C.

### Fluorometry

A 384-well microplate (Corning) was loaded with the reaction volumes and was assayed in a fluorometer (Varioskan Lux, Thermo) to measure green fluorescence (excitation: 495/5 nm, emission: 520/12 nm for fluorescein-labeled oligos; excitation: 466/5 nm, emission: 503/12 nm for the Baby Spinach RNA aptamer).

### Gel Electrophoresis

Samples were loaded on a 10% polyacrylamide gel (acrylamide:*N*,*N*′-methylenebis(acrylamide) ratio of 39:1), which was run for 2.5 h at 75 mA in a cold room. The gel was first stained with ethidium bromide and then with AgNO_3_. The GeneRuler Ultra Low Range DNA ladder (10–300 bp, Thermo) was used as an electrophoresis marker.

## Supporting Information Available

Extended Materials and Methods, Figures S1–S6, and Table S1 (PDF)
