# Supplementary material for: CRISPR-Mediated Strand Displacement Logic Circuits with Toehold-Free DNA
Source: ACS Synth Biol. 2021 Apr 26;10(5):950–6. doi: 10.1021/acssynbio.0c00649 (PMC8489798; doi:10.1021/acssynbio.0c00649)
Supplement: Supplementary file 1 — sb0c00649_si_001.pdf [file sb0c00649_si_001.pdf]

Supplementary Information

CRISPR-mediated strand displacement logic circuits with toehold-free DNA

Roser Montagud-Martínez[a], María Heras-Hernández[a], Lucas Goiriz[a], José-Antonio Daròs[b], and Guillermo Rodrigo[a]

a) I2SysBio, CSIC – Universitat València, Cat. Agustín Escardino 9, 46980 Paterna, Spain
b) IBMCP, CSIC – Universitat Politècnica València, Av. Naranjos s/n, 46022 Valencia, Spain

Materials and Methods

Reagents. The strand displacement reactions were carried out in 1x TAE buffer pH 8.5 (Invitrogen, Thermo ref. AM9869) supplemented with 12.5 mM $MgCl_2$ (Merck, ref. M2393) and 0.05% Tween 20 (Merck, ref. P9416). The different oligos were chemically synthesized by Sigma (now Merck) or IDT. For CRISPR-mediated strand displacement, the *S.p.* Cas9 H840A Nickase V3 (IDT, ref. 1081065) was exploited. To implement the different systems, additional enzymes were used: Proteinase K (Invitrogen, Thermo ref. AM2546), RNase A (Invitrogen, Fisher ref. 12091021), RNase H (Ambion, Fisher ref. AM2292), and RNase Inhibitor (Applied, Fisher ref. 10615995). The chemical compounds PMSF (Thermo, Fisher ref. 10485015) and DFHBI (Merck, ref. SML1627) were also used. For *in vitro* transcription, the TranscriptAid T7 High Yield Transcription kit (Thermo, ref. K0441) was employed.

Reactions. For the prehybridization of the complex species, samples were heated at 95 ºC for 2 min and then cooled slowly to 25 ºC. All sgRNAs were produced in house by *in vitro* transcription and then purified in a column (RNA clean and concentrator kit, Zymo, ref. R1018). Here, 0.2 or 0.5 mL tubes were used for all reactions (Sarstedt). Each reaction was performed in triplicate.

In the case of the initial molecular converter, the input species *IN1* (dsDNA) was introduced at 62.5 nM. The CRISPR reaction was performed during 1 h, with *GUI1* (sgRNA) at 1 µM or 300 nM and Cas9n at 300 nM. The final volume of the reaction was 40 µL, and all steps were carried out isothermally at 37 ºC in a thermocycler (Eppendorf). To assess the effect of *BUF1* or *BUF1:OUT1b*, the CRISPR reaction was incubated for 1 h at 37 ºC, with the same concentrations as before. For the close-loop amplifier, the species $IN1^+$ (ssDNA) was introduced at 62.5 nM to interact with $IN1^-$*:OUT1*, present in the medium also at 62.5 nM. *GUI1* and Cas9n were both present at 300 nM. The final volume of the reaction was 40 µL, which occurred isothermally at 37 ºC during 1 h in a thermocycler (Eppendorf).

In the case of the cascade (CRISPR-mediated strand displacement, plus toehold-mediated strand displacement, plus *in vitro* transcription), the input species *IN2* (dsDNA) was introduced at 125 nM. The CRISPR reaction was performed during 1 h, with *GUI2* (sgRNA) at 600 nM and Cas9n also at 600 nM. At this point, the volume of the reaction was 22.9 µL. The resulting product was treated in the same tube with Proteinase K at 200 µg/mL for 30 min to digest Cas9n. Then, PMSF was added at 1 mM to inactivate Proteinase K, and the reaction was incubated for additional 30 min. Next, RNase A was added at 20 µg/mL to digest the sgRNA, incubating for 30 more min. Subsequently, the species *BUF2:BUF2b* and *BUF3:OUT3* were added at 62.5 nM and 15.6 nM, respectively, and the reaction was incubated for 1 h to let the toehold-mediated strand displacement to occur. At this point, the volume of the reaction was 32 µL. Then, the RNase Inhibitor was added at 4 U/µL to prevent the degradation of the following RNA product by RNase A. To carry out the T7 RNA polymerase-based *in vitro* transcription, the element *BUF3b* was introduced at 7 nM, which contains the reverse complementary sequence of the Baby Spinach RNA aptamer, and the reaction was incubated for 30 min. The fluorophore DFHBI was added at 10 µM to monitor the production of the aptamer. The final volume of the

reaction was 55.6 µL, and all steps were carried out isothermally at 37 ºC in a thermoblock (Labnet). In controls to assess the partial process without CRISPR reaction, other concentrations for the species were used (indicated where it applies).

In the case of the combinatorial device (AND gate), the input species *IN4* (ssDNA) was added at 62.5 nM and the input species *IN5* (regular dsDNA) at 125 nM. The CRISPR reaction was performed during 1 h, with *GUI5* (sgRNA) at 600 nM and Cas9n also at 600 nM. At this point, the volume of the reaction was 32.6 µL. The resulting product was treated in the same tube with Proteinase K at 200 µg/mL for 30 min. Then, PMSF was added at 1 mM and the reaction was incubated for additional 30 min. Next, RNase A was added at 20 µg/mL, incubating for 30 more min. The AND gate element (*BUF4:BUF5:OUT6*) was then added at 62.5 nM and the reaction was incubated for 1 h to let the toehold-mediated strand displacement to occur. The final volume of the reaction was 40 µL, and all steps were carried out isothermally at 37 ºC in a thermocycler (Eppendorf).

Fluorometry. The microplate (384 wells, black, clear bottom; Corning) was loaded with the reaction volumes from tubes (40 µL/well, except after *in vitro* transcription, with 55.6 µL/well). The microplate was assayed in a fluorometer (Varioskan Lux, Thermo) to measure green fluorescence (excitation: 495/5 nm, emission: 520/12 nm for fluorescein-labelled oligos; excitation: 466/5 nm, emission: 503/12 nm for the Baby Spinach RNA aptamer). The measurement time was 100 ms, with automatic range and top optics. The value of underlying fluorescence, corresponding to the reaction buffer, was subtracted to correct the signals. In some cases, the value of background fluorescence corresponding to the quenched fluorophore was also subtracted.

Gel Electrophoresis. In this case, reactions were performed in 20 µL with double concentration. Loading buffer was added to bring the mix to 5% glycerol, 1 mM EDTA, 0.0025% bromophenol blue, and 0.0025% xylene cyanol. Samples were loaded on a 10% polyacrylamide gel (acrylamide:*N,N'*-methylenebisacrylamide ratio of 39:1) of 140 x 130 x 2 mm, which was run for 2.5 h at 75 mA in a cold room. The gel was first stained with 0.5 µg/mL ethidium bromide by shaking for 15 min and then with $AgNO_3$. The GeneRuler Ultra Low Range DNA ladder (10-300 bp, Thermo) was used as an electrophoresis marker.

Additional displays

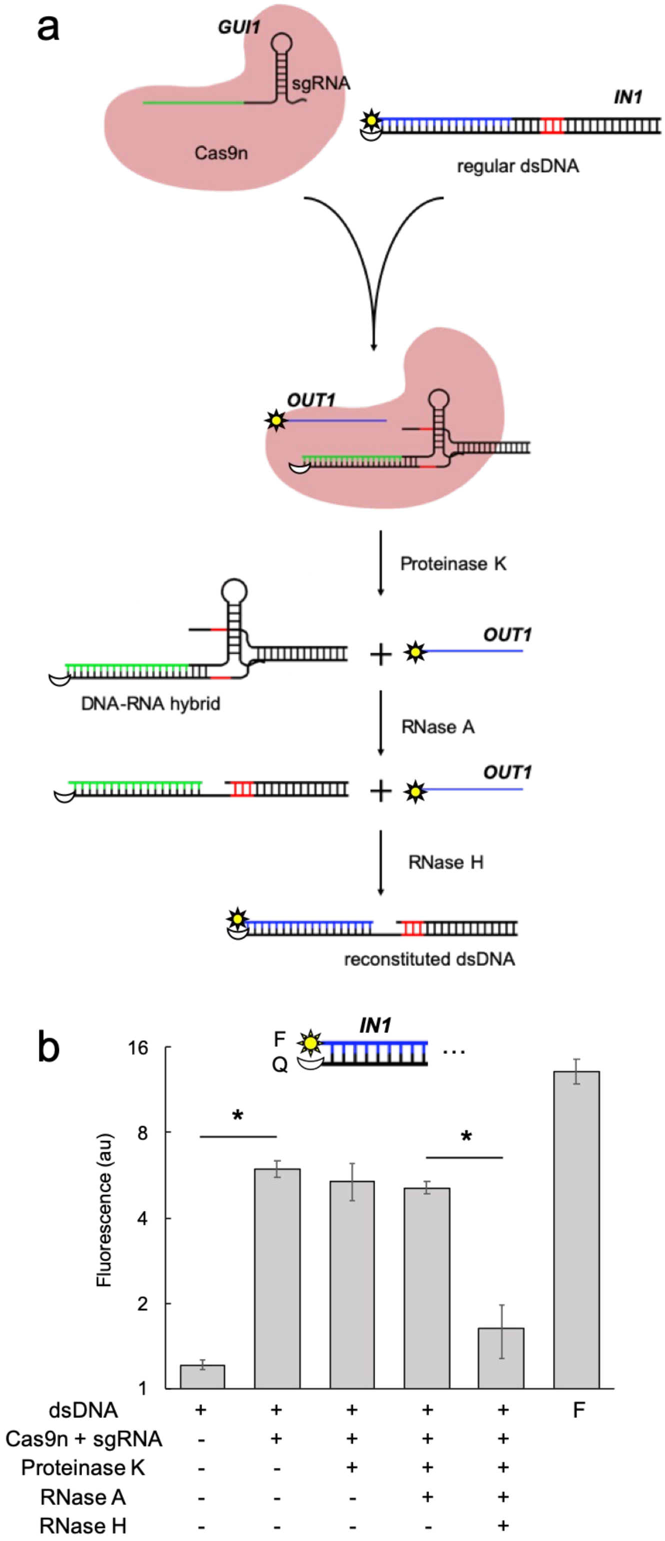

Figure S1. a) Scheme of a nested enzymatic reaction with CRISPR, proteinase K, RNase A, and RNase H by using as input regular DNA (*IN1* species). b) Characterization of the intended strand displacement by using a fluorophore (F) and a quencher (Q). Error bars correspond to standard deviations over replicates (n = 3). *Statistical significance (Welch's t-test, two-tailed $P < 0.05$). Note that the increase of fluorescence upon the CRISPR reaction (second bar) does not entail a release of *OUT1* to the medium, but just a separation from the complementary strand, remaining bound to Cas9n.

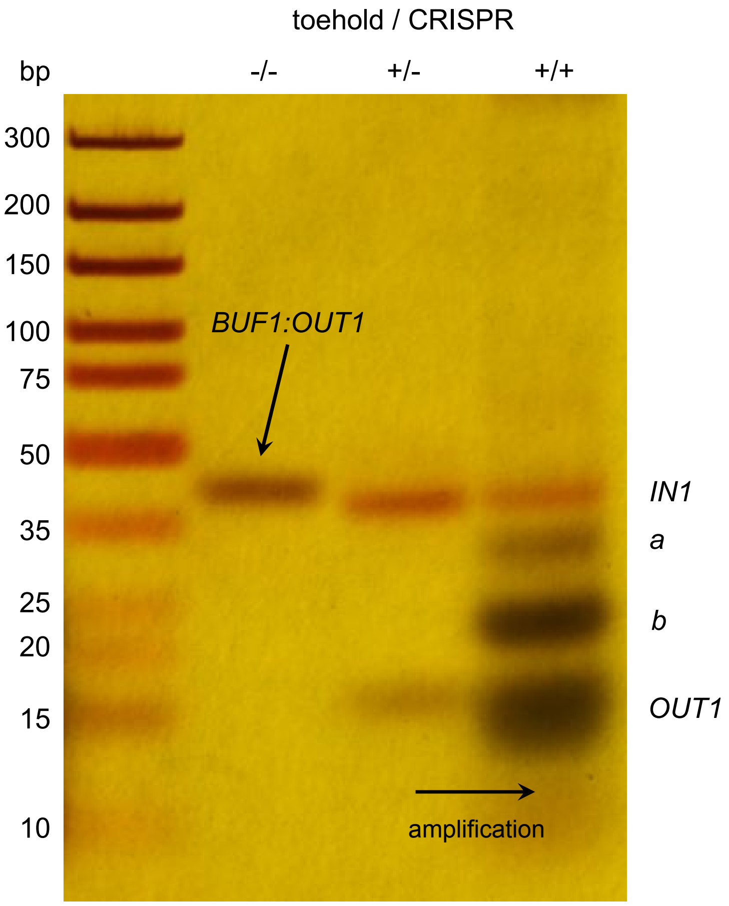

Figure S2. Electrophoretic assay of the close-loop molecular amplifier in which the RNase A treatment is applied. We hypothesized that the band labelled as *a* corresponded to the nicked *IN1* element (written as *IN1n*) and the band labelled as *b* corresponded to the RNA-DNA hybrid species formed by *IN1n* and the protospacer of *GUI1*. We attribute the presence of *IN1n* in the lane +/+ to an unbinding of *GUI1* from the target DNA when Cas9n is digested by proteinase K.

Input: ssDNA sequence
Output: Circuit components sequences, circuit score

1 Let $n = length\ of\ input\ sequence$
2 Let $Root\ =\ the\ concatenation\ of\ input\ sequence\ and\ output\ sequence$
3 Define $sensor$ as the reverse complementary sequence of the bases ranging from the positions 1 to $n + 3$ (included) of $Root$.
4 Define $transducer$ as the bases ranging from the position 7 to the end of $Root$
5 Define $clamp$ as the base reverse complementary sequence of the bases ranging from the positions $n - 5$ to the end of $Root$
6 Let $circuit$ be the set formed by $sensor$, $transducer$ and $clamp$
7 Let $P_1 = \frac{e^{-\beta\Delta G_{\text{input:sensor}}}}{e^{-\beta\Delta G_{\text{input:sensor}}} + e^{-\beta\Delta G_{\text{sensor:transducer}}}}$ where β is the thermodynamic beta and $\Delta G_{a:b}$ is the minimum free energy of duplex $a{:}\,b$
8 Let $P_2 = \frac{e^{-\beta\Delta G_{\text{transducer:clamp}}}}{e^{-\beta\Delta G_{\text{transducer:clamp}}} + e^{-\beta\Delta G_{\text{clamp:output}}}}$
9 Let $P_3 = \frac{e^{-\beta\Delta G_{\text{sensor:transducer}}}}{e^{-19\beta\Delta G_{\text{bp}}}}$ where ΔG_{bp} is the average MFE of each base pair involved in the structure
10 Let $P_4 = \frac{e^{-\beta\Delta G_{\text{clamp:}output}}}{e^{-L\beta\Delta G_{\text{bp}}}}$ where L is the length of $output\ sequence$
11 Let $T = number\ of\ "."\ in\ toehold\ position\ of\ sensor{:}\ transducer\ duplex\ structure\ in\ in\ dot - bracket\ notation$
12 Define $score = P_1P_2P_3P_4\left(\frac{6-T}{6}\right)$
13 **for** 10^5 *iterations* **do**
14   Mutate a random base of a random sequence in $circuit$
15   Compute new $circuit\ score$ following lines 7 to 12
16   **if** $new\ circuit\ score \geq initial\ circuit\ score$ **then**
17   Set new $circuit$ as initial $circuit$
18   **else**
19   Compute $M = e^{-\beta_M^\circ \delta^t (initial\ circuit\ score\ -\ new\ circuit\ score)}$ where $\beta_M^\circ \approx 1100$, $\delta = 1 + 7 \cdot 10^{-5}$ and $t\ =\ current\ iteration$.
20   **if** $[random\ number\ between\ 0\ and\ 1] < M$ **then**
21   Set new $circuit$ as initial $circuit$
22   **end**
23   **end**
24 **end**
25 **Return** $circuit,\ score$

Figure S3. Pseudocode of an algorithm for the automated sequence design of a molecular converter of two arbitrary sequences. The final set of sequences results from an *in silico* evolutionary process that selects the species with the highest objective function. To avoid entrapment in local maxima, a Metropolis approach is used.

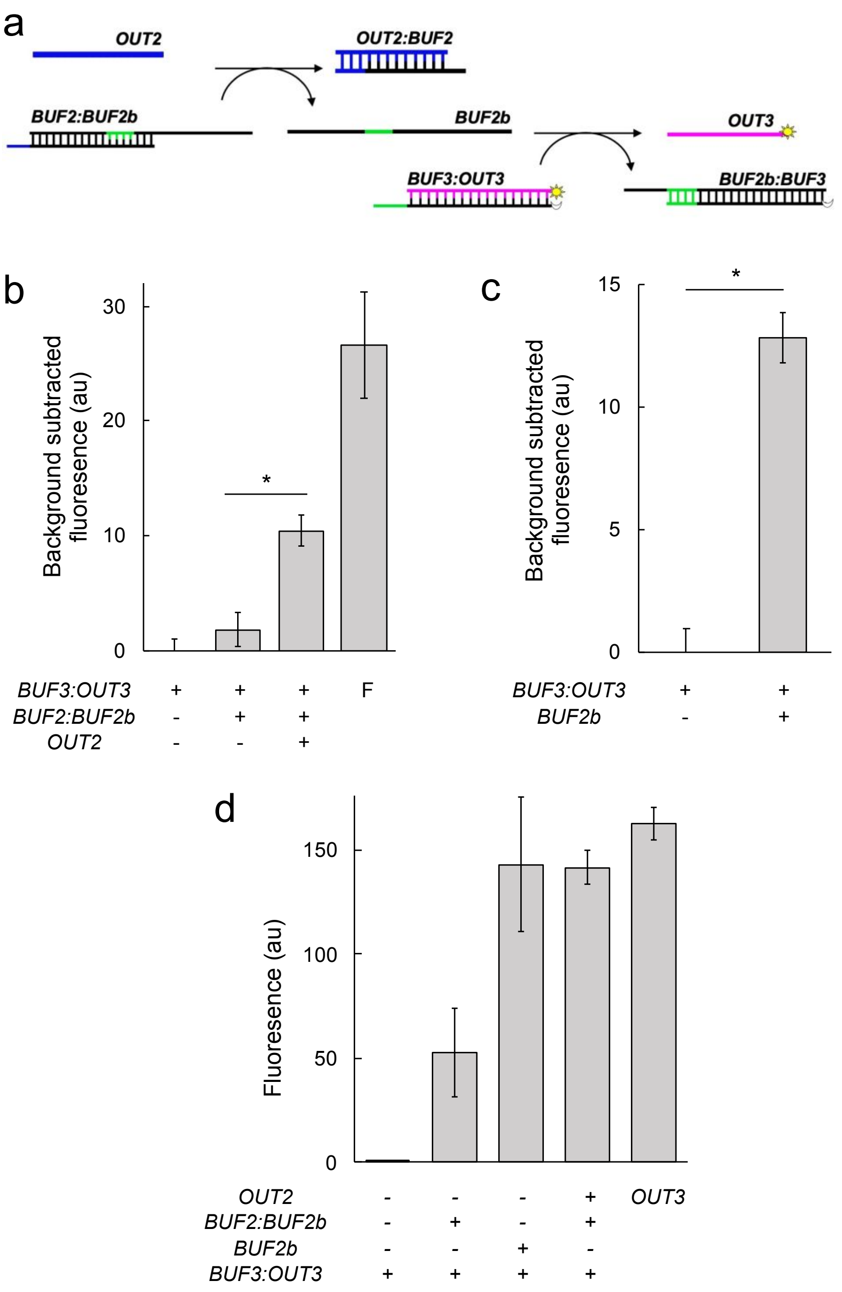

Figure S4. a) Detailed schematics of the toehold-mediated strand displacement to interconvert two arbitrary ssDNA species. b) Characterization of a toehold-mediated strand displacement reaction by using a fluorophore and a quencher, taking as input the ssDNA species *OUT2*. c) Characterization of a toehold-mediated strand displacement reaction, taking as input the ssDNA species *BUF2b*. Error bars correspond to standard deviations over replicates (n = 3). In these experiments, all species were at 1 µM and the reaction was at 37 ºC for 1 h (volume of 5 µL). d) Characterization of the *in vitro* transcription of a fluorescent RNA aptamer from the release of the ssDNA species *OUT3* in a toehold-mediated strand displacement reaction. Error bars correspond to the ranges (n = 2). *Statistical significance (Welch's t-test, two-tailed P < 0.05). In these experiments, all species were at 250 nM (expect for *BUF3b*, of which 1 µg was used) and the reaction was at 37 ºC for 1 h for strand displacement (volume of 5 µL) and 30 min for *in vitro* transcription (volume of 20 µL).

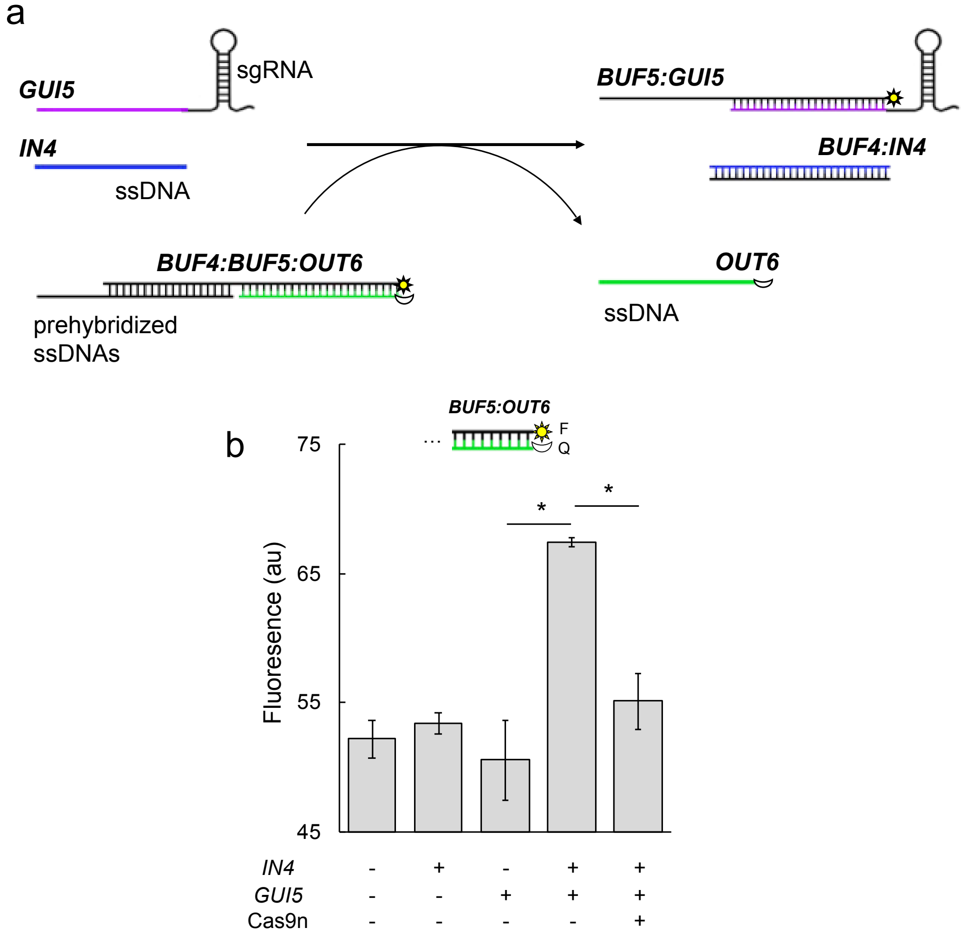

Figure S5. a) Scheme of the AND gate activated by the ssDNA *IN4* and the sgRNA *GUI5*. b) Characterization of the intended strand displacement by using a fluorophore (F) and a quencher (Q). Error bars correspond to standard deviations over replicates (n = 3). *Statistical significance (Welch's t-test, two-tailed P < 0.05). In these experiments, *GUI5* and Cas9n were at 125 nM. The presence of Cas9n prevents the activation by *GUI5*.

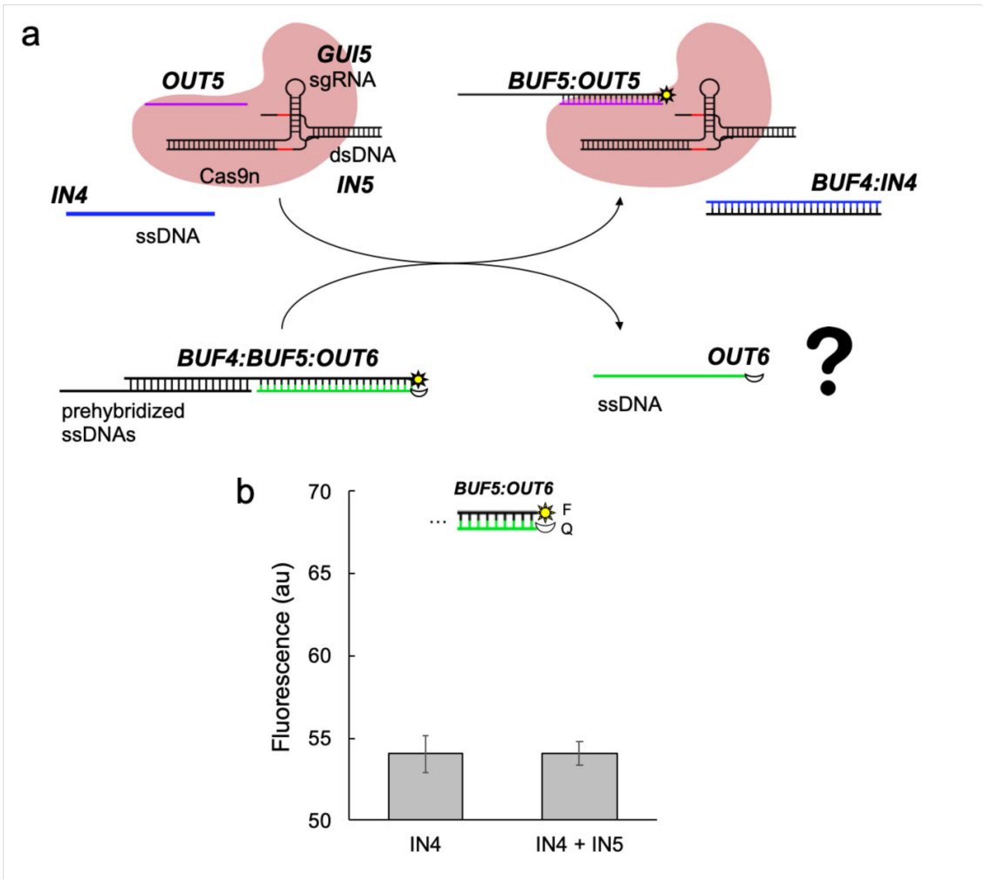

Figure S6. a) Scheme of the potential interaction between *BUF5:OUT6* and *OUT5* in the CRISPR complex. b) Characterization by using a fluorophore (F) and a quencher (Q) showing no strand displacement. Error bars correspond to standard deviations over replicates (n = 3). When *BUF4:BUF5:OUT6* (62.5 nM) is not at a higher concentration than the CRISPR complex (125 nM), the reaction does not progress. If *BUF4:BUF5:OUT6* were at a much higher concentration, the reaction might progress, but the dynamic range of the response would be limited by the concentration of the CRISPR complex.

Table S1. Sequences of all nucleic acid species used in this work.

Id	Type	Sequence
IN1	dsDNA	5′-GGCTAAAGAGGAAGAGGACATGGTGAATTCGTAACT<br>3′-CCGATTTCTCCTTCTCCTGTACCACTTAAGCATTGA
IN1b	dsDNA	5′-GGCTAAAGAGGAAGAGGACATGG<br>3′-CCGATTTCTCCTTCTCCTGTACC
GUI1	sgRNA	5′-GGCUAAAGAGGAAGAGGACAGUUUUAGAGCUAGAAAUAGCAAGUU AAAAUAAGGCUAGUCCGUUAUCAACUUGAAAAAGUGGCACCGAGUCGG UGCUUUU
GUI1b	sgRNA	5′-GGCUAAAGAGGAAGAGGACAGUUUUAGAGCUAGAAAUAGCAAGUU AAAAUAAGGCUAGUCCG
OUT1	ssDNA	5′-GGCTAAAGAGGAAGAGG
BUF1	ssDNA	5′-CCATGTCCTCTTCCTCTTTAGCC
OUT1b	ssDNA	5′-AGAGGAAGAGGACATGG
IN1⁺	ssDNA	5′-GGCTAAAGAGGAAGAGGACATGGTGAATTCGTAACT
IN1⁻	ssDNA	5′-AGTTACGAATTCACCATGTCCTCTTCCTCTTTAGCC
IN2	dsDNA	5′TGGAGTGTGACAATGGTGTTTGACATGGTCTAACGCATTCGTAACC<br>3′ACCTCACACTGTTACCACAAACTGTACCAGATTGCGTAAGCATTGG
GUI2	sgRNA	5′GGUGGAGUGUGACAAUGGUGUUUGACAGUUUUAGAGCUAGAAAUAG CAAGUUAAAAUAAGGCUAGUCCGUUAUCAACUUGAAAAAGUGGCACCG AGUCGGUGCUUUU
OUT2	ssDNA	5′TGGAGTGTGACAATGGTGTTTG
BUF2	ssDNA	5′CGCCAAACACCATTGTCACACTCCA
BUF2b	ssDNA	5′GTGACAATGGTGTTTGGCGCTAATACGACTTACTATAGG
BUF3	ssDNA	5′CCTATAGTGAGTCGTATTAGCGCCAAACA
OUT3	ssDNA	5′GCGCTAATACGACTCACTATAGG
BUF3b	ssDNA	5′GGAGCTCACACTCTACTCAACAGTAGCGAACTACTGGACCCGTCCT TCACCTATAGTGAGTCGTATTAGCGC
Baby Spinach	RNA aptamer	5′GGUGAAGGACGGGUCCAGUAGUUCGCUACUGUUGAGUAGAGUGUGA GCUCC
IN4	ssDNA	5′-GGCTTTCACATTACTTTTGCTGCCTTACGAGTCTTC
IN5	dsDNA	5′-TTCCAAGAGTGATATGCCAATACAAACCACGAAGACACATGGTCT AACGCATTCGTAACC<br>3′-AAGGTTCTCACTATACGGTTATGTTTGGTGCTTCTGTGTACCAGA TTGCGTAAGCATTGG

GUI5	sgRNA	5'-GGUUCCAAGAGUGAUAUGCCAAUACAAACCACGAAGACACAGUUUUAGAGCUAGAAAUAGCAAGUUAAAAUAAGGCUAGUCCGUUAUCAACUUGAAAAAGUGGCACCGAGUCGGUGCUUUU
OUT5	ssDNA	5'-TTCCAAGAGTGATATGCCAATACAAACCACGAAGAC
BUF4	ssDNA	5'-GAAGACTCGTAAGGCAGCAAAAGTAATGTGAAAGCC
BUF5	ssDNA	5'-CACATTACTTTTGCTGCCTTACGAGTCTTCGTGGTTTGTATTGGCATATCACTCTTGGAA
OUT6	ssDNA	5'-TTCCAAGAGTGATATGCCAATACAAACCAC
